# The effect of preload on Doppler systolic time in a large emergency department cohort with suspected sepsis

**DOI:** 10.1186/s40635-026-00987-1

**Published:** 2026-10-12

**Authors:** Sarah Atwi, Jared Nunnally, Kristen Ugale, So Mi Ko, Jaclyn Bond, Kimberly Boozer, Raymond J. Bautista, Catalina Torres, Matthew Sedillo, Daniel Lachner-Piza, Tracy Savery, Melissa Hebscher, Joseph K. Eibl, Jon-Emile S. Kenny, Korbin Haycock

**Affiliations:** 1 Flosonics Medical, 325 W. Front Street, Toronto, ON M5V2Y1 Canada; 2 Department of Emergency Medicine, Riverside University Health System Medical Center, Moreno Valley, CA USA; 3 Northern Ontario School of Medicine, 935 Ramsey Lake Rd, Sudbury, ON P3E2C6 Canada; 4 Health Sciences North Research Institute, 56 Walford Rd, Sudbury, ON P3E 2H2 Canada; 5 Department of Emergency Medicine, Loma Linda University Medical Center, Loma Linda, CA USA

**Keywords:** Left ventricular ejection time, Corrected flow time, Carotid artery, Doppler ultrasound, Wearable technology, Functional hemodynamic monitoring, Fluid responsiveness

## Abstract

**Background:**

Change in the corrected flow time of the common carotid artery (ccFT_Δ_) during a preload challenge (PC) is a surrogate for stroke volume change (SV_Δ_) to infer preload responsiveness (P_R_).

**Methods:**

This is a retrospective cohort study comprising patients with suspected sepsis in a large, publicly funded, academic emergency department; no patient health information is linked to the Doppler assessments. Carotid Doppler spectrograms from a wireless, wearable critical care Doppler ultrasound system are analyzed for ccFT_Δ_ during a PC (either infusion of 300 mL of crystalloid over 3 min or a Trendelenburg maneuver). The primary objectives of this study are to describe the proportion of P_R_ patients by previously described ccFT_Δ_ thresholds (i.e., both 4 and 7 ms ccFT_Δ_ ), describe time-to-peak (TTP) ccFT_Δ_, and evaluate the ability of baseline ccFT to detect ccFT_Δ_ during PC, using both the equations of Wodey and Bazett. Comparisons of related outcomes were adjusted for multiple comparisons using the Holm procedure, baseline ccFT discrimination was quantified using area under the receiver operating characteristic curve, and TTP was estimated using patient-level bootstrap resampling. Threshold-agnostic ccFT_Δ_ trajectories were additionally explored using unsupervised k-means clustering of patient-level temporal responses for both correction equations.

**Results:**

A PC was attempted in 4367 patients and an adequate ccFT_Δ_ was collected from 4,128 patients (95%). In total, 982,937 Doppler cardiac cycles are included. The median ccFT_Δ_ was 7.3 milliseconds (ms). 51.9% of patients had a ccFT_Δ_ ≥ 7 ms, while 68.6% had a ccFT_Δ_ ≥ 4 ms by the equation of Wodey. For Bazett, these fractions were 61.2% and 73.5%, respectively. For patients identified as P_R_ based upon either a 4 ms or 7 ms ccFT_Δ_ threshold, the time-to-peak ccFT_Δ_ was 27.5 s for both equations. Baseline absolute ccFT did not discriminate P_R_ from P_UR_ patients under both correction formulae (AUROC 0.56–0.57).

**Conclusions:**

To our knowledge this is the largest reported dataset of ED patients with suspected sepsis receiving P_R_ assessments by Doppler ultrasound, comprising nearly 1 million Doppler cardiac cycles obtained from over 4100 ED patients. Both 7 ms and 4 ms ccFT_Δ_ thresholds described clinically-accepted rates of P_R_. The TTP was roughly 30 s into PC and baseline ccFT did not accurately detect ccFT_Δ_ during PC. Finally, a hypothesis-generating analysis identified four distinct ccFT_Δ_ trajectories during PC which may represent unique preload response patterns during P_R_ testing.

**Supplementary Information:**

The online version contains supplementary material available at https://doi.org/10.1186/s40635-026-00987-1.

## Background

The duration of mechanical systole is measured as the time from the onset of systole to its offset [1]; this can be obtained from either the left ventricular outflow tract (LVOT) or from common carotid artery Doppler pulses as they are interchangeable [2]. The duration of mechanical systole is the time that the aortic valve is open and ejecting blood and is a long-established measure of left ventricular function combining elements of stroke volume (SV), afterload and contractility [1]. When afterload and contractility are relatively constant, changing systolic time and stroke volume (SV_Δ_) are directly proportional. This is true primarily during preload change, for example, during a passive leg raise, Trendelenburg or rapid fluid challenge [3–5]. With this, change in systolic duration in response to preload augmentation helps detect preload responsiveness (P_R_) – or a significant SV_Δ_ – a resuscitation paradigm that significantly reduces mortality in sepsis [6].

There are many tests and measurement approaches to P_R_ that can be tailored based upon patient phenotypes [7]. In our emergency department (ED), rapid response team (RRT) and sepsis emergency response team (SERT), we employ a recently developed wireless, wearable critical care Doppler ultrasound system that continuously measures the time of systole from the carotid artery corrected for heart rate (i.e., the carotid corrected flow time, ccFT) during preload augmentation as a test for P_R_ [8–15]. Here we present a descriptive analysis of this growing dataset with the following objectives: (1) describe the median ccFT_Δ_ as a measure of P_R_ for over 4100 septic ED patients; (2) describe the trajectory of ccFT_Δ_ classified by responsiveness, cardiac cycle regularity, and unsupervised cohorts; and (3) describe the interaction between baseline absolute ccFT and P_R_ based upon both 4 and 7 ms ccFT_Δ_ thresholds. We hypothesize that a patient’s baseline, static, absolute ccFT will not predict change in ccFT (ccFT_Δ_) during preload augmentation.

## Methods

### Study population

This is a retrospective cohort study comprising over 4367 patients in whom severe sepsis was identified in a large, publicly funded, academic ED in southern California. This dataset includes patients evaluated between January 2023 and May 2026; though clinical data was captured for all patients, for this analysis, no patient health information is linked to the Doppler assessments.

### Ethical approval

The study protocol (Pro00085445) was approved by the Advarra Institutional Review Board on May 24th, 2026, with a determination of exemption from Investigational Device Exemption (IDE) regulations and a waiver of patient informed consent to analyze de-identified data.

### Inclusion and exclusion criteria

At our institution, all patients in the ED or general medical floor in whom changes in vital signs or laboratory values trigger a Best Practice Alert (BPA) in the electronic medical record and are identified to have severe sepsis (i.e., the presence of at least two SIRS criteria, an identified or presumed infectious source and end-organ dysfunction) are seen by a dedicated nursing-led rapid response team or sepsis emergency response team (SERT) [14]. As described previously [14, 16, 17], our SERT is composed of RNs with dedicated point of care ultrasound (POCUS) and sepsis resuscitation training. This training begins with POCUS examinations accompanied by an experienced SERT preceptor followed by physician review of at least 10 examinations. The trainee must demonstrate aptitude to pass training and certify competency. Afterwards, SERT RNs maintain competency by exhibiting the POCUS skills with the lead physician (K.H.) instructor yearly. To illustrate the effectiveness of our training program, we conducted a prospective observational study revealing that ED physicians agreed with SERT RN POCUS assessments in virtually all cases [16, 17]. The SERT registered nurse (RN) employs bedside ultrasound and a device to assess P_R_ and/or fluid tolerance [12, 18, 19] including use of the wireless, wearable 4 MHz, continuous wave, F.D.A. cleared critical care ultrasound system (Flosonics Medical, Sudbury, Canada).

Clinical exclusion criteria for using the wearable Doppler ultrasound are known, severe (> 60%), bilateral carotid artery stenosis, inability to cooperate with a hemodynamic intervention (e.g., hyperactive delirium), known severe aortic or mitral valve disease, age less than 18 years or contraindication for augmenting preload.

### Doppler system and measures

**Fig. 1:**
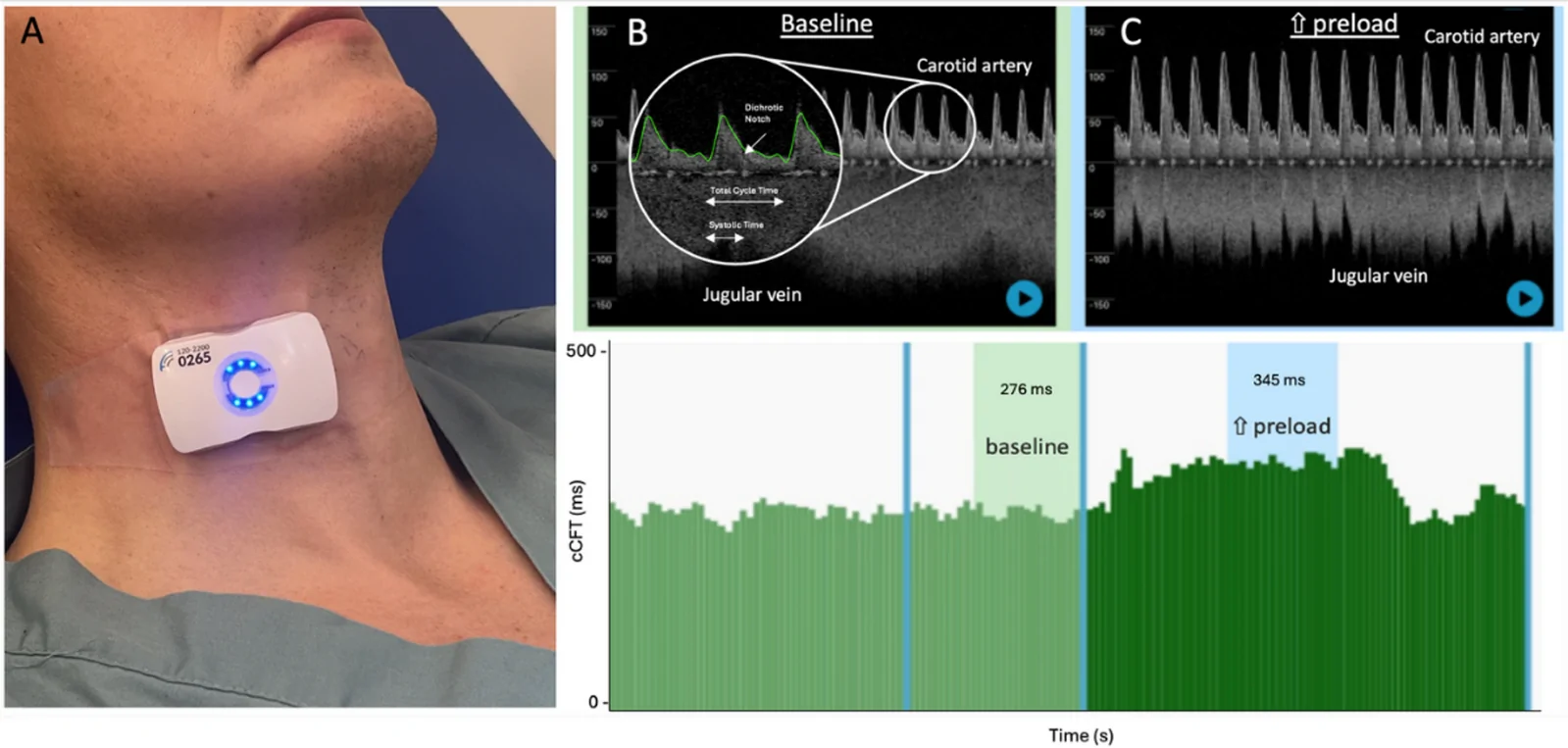
Description of the wearable critical care Doppler ultrasound device and interface used for data collection. **A**: The wearable critical care Doppler ultrasound device adhered to the neck of a sample patient. **B** and **C**: The carotid Doppler spectrogram and features used to calculate ccFT values calculated per cardiac cycle on device interface (inset) in baseline (**B**) and during PC (**C**)

The wireless wearable Doppler ultrasound is a U.S. F.D.A. cleared critical care ultrasound (CCUS) system that auto-calculates the duration of Doppler systole and its change during preload augmentation. The device is shown in Fig. 1A along with its graphical user interface and features of the spectrogram used to calculate the ccFT. Individual cardiac cycles were excluded if features were not detectable due to signal drop, artifact interference, low signal-to-noise ratio, irregular pulse morphology, or poor usability of derived waveform features. The ccFT is calculated for each cardiac cycle and displayed as shown in Fig. 1 by the equation of Wodey (Eq. 1) [3, 4]; though a recent evaluation also used the equation of Bazett (Eq. 2) [5, 20]. In all subjects the common carotid Doppler pulse was detected by the displayed spectrogram and audible Doppler shift consistent with the common carotid artery. Once obtained, the wearable ultrasound device is adhered in place.

1 $$\:\:{\mathrm{c}\mathrm{c}\mathrm{F}\mathrm{T}}_{\mathrm{W}\mathrm{o}\mathrm{d}\mathrm{e}\mathrm{y}}=\mathrm{F}\mathrm{l}\mathrm{o}\mathrm{w}\mathrm{T}\mathrm{i}\mathrm{m}\mathrm{e}+1.29\mathrm{*}(\mathrm{H}\mathrm{e}\mathrm{a}\mathrm{r}\mathrm{t}\mathrm{R}\mathrm{a}\mathrm{t}\mathrm{e}-60)$$

2 $$\:{\mathrm{c}\mathrm{c}\mathrm{F}\mathrm{T}}_{Bazett}=\mathrm{F}\mathrm{l}\mathrm{o}\mathrm{w}\mathrm{T}\mathrm{i}\mathrm{m}\mathrm{e}\mathrm{*}1/\sqrt{60/HeartRate}$$

### Preload challenge

All patients receive a preload challenge (PC) as either a rapid fluid challenge of 100 mL/min over 2–3 min or Trendelenburg positioning from 30 degrees head up to 10–20 degrees head below the horizontal for at least 90 s; PC type is decided by the treating clinician. The rate and volume of the rapid fluid challenge was derived from a systematic review and meta-analysis [21] and a crystalloid pharmacokinetic study using real-time Doppler ultrasonography as the response measure. In the pharmacokinetic study [22], the authors administered normal saline in 50 mL aliquots over 30 s and found that the optimal delivered volume to detect P_R_ was 250 mL. At our institution, Trendelenburg position is contraindicated at the discretion of the treating clinician. Conditions which preclude this maneuver are when it is not clinically safe to transiently increase intracerebral pressure, if there is concern for venous thrombosis or pulmonary embolus, when there is a significant aspiration risk and/or if there is intra-abdominal hypertension (including a gravid uterus), which can lead to false negative P_R_ results because of impaired abdominal venous return [23].

To determine P_R_, carotid Doppler spectrograms are analyzed for ccFT_Δ_, calculated as the difference between cardiac cycles averaged during ‘assessment’ (i.e., RFC or head-down) and before assessment (i.e., ‘baseline’). Baseline windows are selected based on a minimum of 15 cardiac cycles, low coefficient of variation, and high density of cardiac cycles. Assessment windows are selected based on a minimum of 20 cardiac cycles, low coefficient of variation, high density of cardiac cycles, and the largest change with baseline. Maximal quality-qualified windows rather than average windows at arbitrary timepoints were chosen in the assessment period to capture all instances of potential physiological response. Sessions that do not meet the criteria for adequate assessment windows were excluded. We chose 4 and 7 ms ccFT_Δ_ thresholds to detect P_R_ based upon 3 previous studies that used bioreactance [3], left ventricular outflow tract velocity time integral from trans-esophageal echocardiography [4] and minimally invasive, auto-calibrated pulse contour analysis [5] as reference standards. These studies reported optimal thresholds between 4 and 7 ms ccFT_Δ_.

### Statistical analysis

Summary statistics were calculated for patient-level ccFT_Δ_ values for each correction method, including the proportion of patients who exceeded the predefined response thresholds of ≥ 4 ms and ≥ 7 ms. Continuous variables were summarized as mean ± standard deviation (SD) when approximately normally distributed and as median [interquartile range (IQR)] when non-normally distributed. Categorical variables were summarized as frequencies and percentages. Distributional assumptions were assessed using the Shapiro-Wilk test and inspection of the observed distributions; baseline vs. assessment stages were compared using Wilcoxon signed-rank test. Proportions of P_R_ were compared between paired Wodey- and Bazett-corrected values using McNemar’s exact test, with Holm correction applied across the two threshold comparisons.

The relationship between mean baseline ccFT (determined from an adequate assessment window described above) and intervention-associated ccFT_Δ_ was assessed at the patient level using Pearson and Spearman correlations calculated separately for Wodey- and Bazett-corrected ccFT. Individual cardiac cycles were not treated as independent observations. Mean baseline ccFT was calculated from the cardiac cycles within each patient’s baseline assessment window, such that each patient contributed a single baseline ccFT value and a single P_R_/P_UR_ classification to each threshold/formula ROC analysis. To determine the ability of baseline ccFT to directly discriminate between P_R_ from P_UR_, we quantified the area under the receiver operating characteristic curve (AUROC) with sensitivity, specificity, and the corresponding baseline ccFT cutoff identified using Youden’s J statistic.

The cohort was divided into regular and irregular. Irregular cardiac cycle rhythm patients were determined based on an automated irregularity detection algorithm. A patient was determined to exhibit irregular beats when more than 25% of beats have a rolling peak-to-peak coefficient of variation above 20%. CcFT measures were compared between patients with regular and irregular cardiac rhythms using two-sided Mann–Whitney U tests. To account for multiple comparisons, p-values were adjusted using the Holm procedure within the prespecified family of irregular-versus-regular comparisons, with a family-wise α of 0.05. Proportions of P_R_ and P_UR_ between irregular and regular patients were compared using a two-sided two-proportion z-tests, with Holm correction applied across the four formula/threshold comparisons.

To illustrate and characterize population-level physiological response temporal trajectory, the beat-level ccFT_Δ_ trajectories (defined as a cardiac cycle ccFT at that point in PC less the mean baseline window ccFT) across responsive and non-responsive patients (determined for each threshold and correction method) were visualized on a common temporal grid referenced to the beginning of assessment and assigned to 5-second bins up to 120 s. Across-session means and 95% confidence intervals were computed for each bin. Additionally, between-patient variability underlying the population-level trajectories was explored in a supplementary analysis in which cardiac cycles were first averaged within each patient and 5-second time bin. The median and interquartile range of these patient-level values were then calculated across patients within each time bin and displayed.

The first sustained time-to-peak (TTP) in ccFT following PC was estimated by patient-level bootstrap resampling. For each iteration (*n* = 1,000), patients were resampled with replacement to the original group size, all contributing beats were pooled, binned into 5-second time-elapsed windows referenced to the start of PC, and averaged to produce a bootstrapped mean ccFT_Δ_ trajectory. TTP was defined as the bin center of the first local maximum occurring at least 10 s after intervention onset, where the local peak and the subsequent two bins all remained at ≥ 90% of the local peak value (a sustained plateau of 15 s). A local-peak definition was used to identify the first true response and avoid sensitivity to late-window drift in the trajectory. Median and 95% confidence intervals are reported.

To explore the threshold agnostic trajectories of ccFT response to PC, a k-means clustering approach was used. K-means clustering requires complete, equal-length feature vectors, residual missing values occurring at the leading or trailing edges of otherwise eligible trajectories were filled using the nearest observed value (backward or forward filling, respectively). Therefore, for each patient, beat-level ccFT_Δ_ was linearly interpolated onto a common 0–120 s time grid in 5-second steps, referenced to the start of PC. To limit the influence of the interpolation procedure, sessions flagged as irregular, with fewer than 10 valid beats, less than 50% grid coverage (< 60 s PC duration), or with more than 30% missing values after interpolation were excluded. Remaining missing values were forward and backward filled, and trajectories were smoothed using a Savitzky-Golay filter (15 s smoothing window). K-means clustering was then applied (k = 2 to 8) and evaluated both within-cluster sum-of-squares (elbow) and average silhouette score; the optimal k was selected by elbow inflection and supported by interpretability of physiology. Post-hoc, phenotypes were assigned to each cluster based on their average trajectory shapes. Phenotype peak ccFT_Δ_ and TTP (as previously defined) were estimated by session-level resampling (1,000 iterations) within each cluster.

## Results

### Study population

**Fig. 2:**
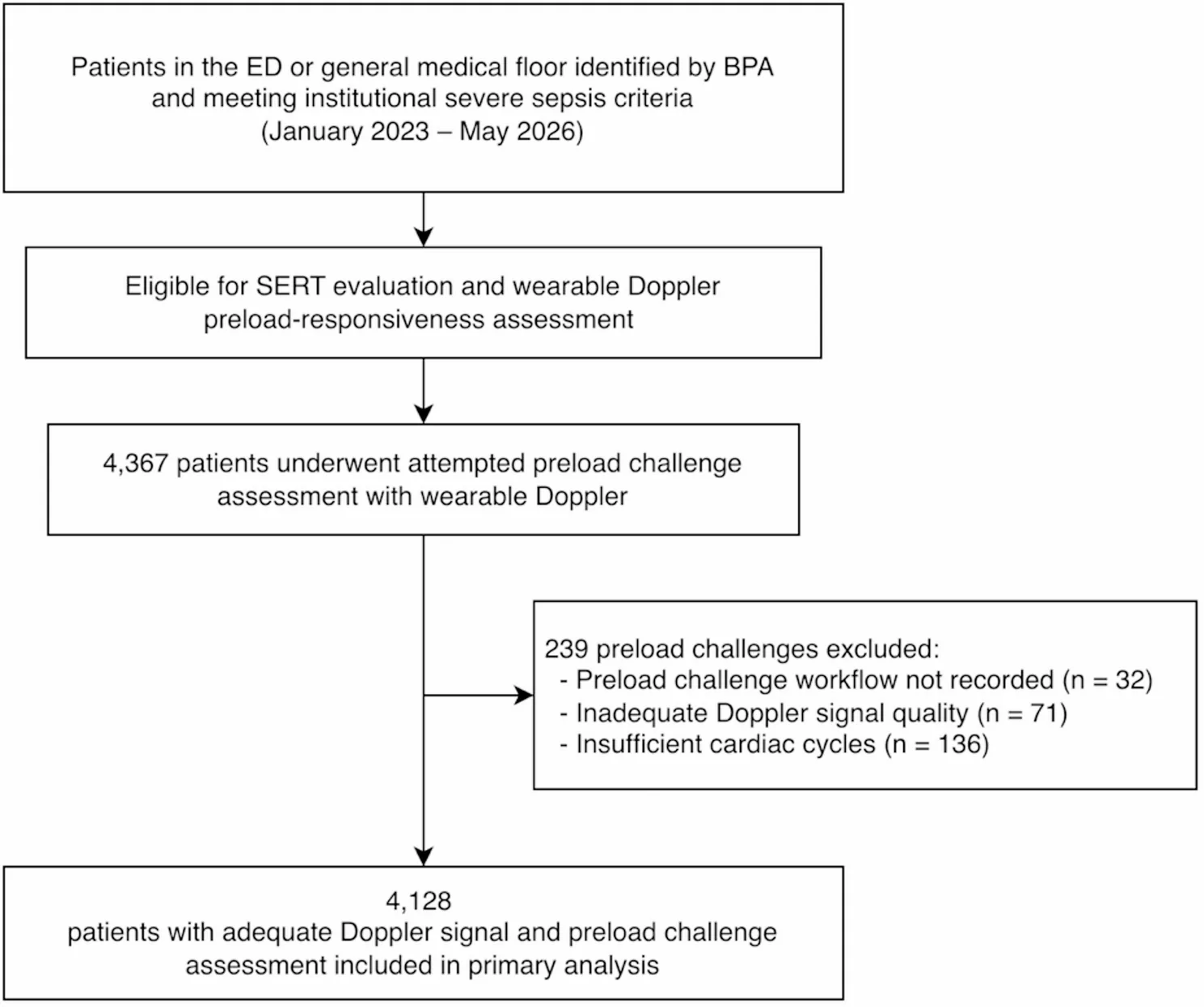
Flow diagram summarising exclusions and reasons for inadequate assessments. Patients were identified through the institutional BPA/severe sepsis pathway and underwent attempted preload-responsiveness assessment with the wearable critical care Doppler ultrasound system. The diagram summarizes exclusions due to inability to obtain an adequate Doppler signal and the final analytic cohort

A PC with the wearable critical care Doppler ultrasound was attempted in 4367 patients. Of these, an adequate ccFT_Δ_ was completed in 4128 patients (i.e., 95%) (Fig. 2). Across all eligible patients, 1,020,238 cardiac cycles were considered. Of these, 37,301 were excluded, leaving 982,937 cardiac cycles for analysis. Mean length of the entire assessment was 131.6 ± 54 s (baseline: 40.3 ± 31 s, PC: 91.5 ± 41 s). Baseline median (IQR) heart rate was 101.4 bpm (87.8-113.5) bpm and ccFT was 281.7 ms (265.9-297.1) using Wodey and 291.9 ms (270.6-312.4) using Bazett. During PC, heart rate dropped to 100.1 bpm (87.1-112.3), however ccFT increased to 286.9 ms (270.7–303.0, Wodey, *p* < 0.01) and 298.7 ms (277.6-319.7, Bazett, *p* < 0.01).

### Distribution of ccFT_Δ_ and preload responsiveness

The median ccFT_Δ_ was 7.3 ms (2.5–12.5) with Wodey, significantly lower than Bazett’s ccFT_Δ_ at 9.64 ms (3.6–16.2, *p* < 0.001). Using Wodey, 51.9% of patients met the responsive threshold of ccFT_Δ_ ≥ 7 ms and 68.6% met the ≥ 4 ms threshold; using Bazett, the corresponding rates were significantly higher at 61.2% and 73.5% (*p* < 0.0001). The distributions of patient-level ccFTΔ are shown in Supplementary Fig. 1.

### Time course of ccFT_Δ_ during preload challenge

**Fig. 3:**
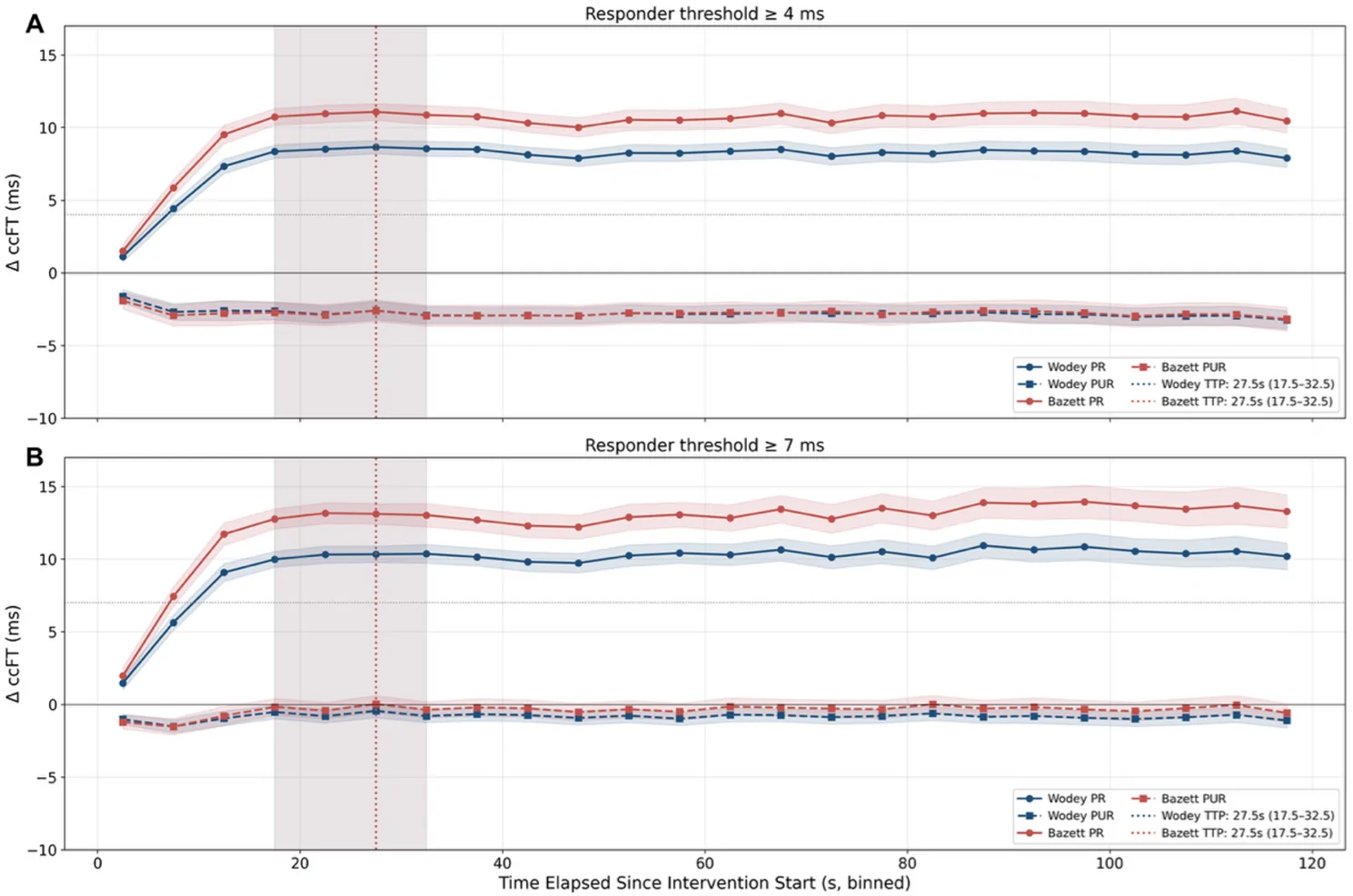
Trajectories of ccFT_Δ_ across time elapsed in P_R_ (circle) and P_UR_ (square) patients based upon a + 4 ms (**A**) and + 7 ms (**B**) for each Wodey (Blue) and Bazett (red). Lines represent the population mean patient-level ccFT_Δ_ within each 5-second time bin, with shading representing the 95% confidence interval around the mean. These confidence intervals quantify precision of the estimated population trajectory and should not be interpreted as the distribution of individual patient responses

For patients identified as P_R_ for both ccFT correction methods and thresholds, the first TTP following PC median (95% CI) was at 27.5 s (17.5–32.5 s) into PC. No sustained rise was observed in unresponsive patients at both thresholds and ccFT correction methods (Fig. 3). While population-level trajectories appear stable, between-patient variability was observed across time bins as illustrated by patient-level median and IQR (Supplementary Fig. 2).

### Diagnostic performance of baseline ccFT

Although significant, baseline ccFT was weakly inversely associated with intervention-associated ccFT_Δ_ using both Wodey (Pearson *r* = − 0.181, Spearman ρ = −0.128, R² = 0.033; both *p* < 0.0001) and Bazett (Pearson *r* = − 0.203, Spearman ρ = −0.160, R² = 0.041; both *p* < 0.0001).

Baseline absolute ccFT discriminated P_R_ from P_UR_ patients only modestly under both correction formulae (AUROC 0.56–0.57 across all four threshold-formula combinations). At the Youden-optimal cutoff, sensitivity ranged 0.59–0.68 and specificity 0.42–0.52 (Wodey ≥ 4 ms: optimal cutoff 283.4 ms, AUROC 0.56; Wodey ≥ 7 ms: 282.9 ms, AUROC 0.56; Bazett ≥ 4 ms: 295.2 ms, AUROC 0.57; Bazett ≥ 7 ms: 300.5 ms, AUROC 0.56).

### Irregular cardiac rhythm subgroup analysis

Of the cohort, 247 patients (6.0%) were classified as having irregular cardiac cycles. As expected, the within-session CV was 2.5-fold higher than in regular patients under both correction formulae (*p* < 0.001), confirming that the two populations are distinct. Irregular patients had similar heart rate at baseline and into PC, while regular patients demonstrated a statistically significant drop that was not clinically significant (i.e., diminished by 1.5 beats per minute). Although absolute ccFT values were lower in irregular patients at both baseline and into PC under both correction formulae, the ccFT_Δ_ did not differ significantly between irregular and regular patients. Among P_R_ patients, the median (95% CI) TTP was 32.5 s (12.5–97.5) using Wodey at both the ≥ 4 ms and ≥ 7 ms thresholds. Using Bazett, the TTP was 27.5 s (12.5–107.5 s) at the ≥ 4 ms threshold and 52.5 s (17.5–97.5 s) at the ≥ 7 ms threshold (Table 1).

**Table 1:** Comparisons of irregular vs. regular patient beats-per-minute (bpm) and corrected carotid flow time (ccFT) using Wodey and Bazett

	Irregular	Regular	Holm-adjusted *p*-value
*n *(%)	247 (6)	3881 (94)	-
Heart Rate (bpm, median (IQR))			
Baseline	100.3 (83.5-113.8)	101.5 (88.3-113.6)	1.0
PC	100.5 (85.8-113.4)	100.0 (87.2-112.2)*	1.0
Baseline ccFT (ms, median (IQR))			
Wodey	269.7 (255.3-289.7)	282.1 (266.8-297.5)	< 0.0001
Bazett	275.6 (256.0-300.4)	292.9 (271.7–313.0)	< 0.0001
PC ccFT (ms, median (IQR))			
Wodey	277.7 (260.2–294.0)*	287.6 (271.8-303.4)*	< 0.0001
Bazett	283.6 (263.6-304.5)*	299.7 (278.7-320.5)*	< 0.0001
ccFT_Δ_ (ms, median (IQR))			
Wodey	7.9 (1.9-14.3)	7.2 (2.5-12.5)	1.0
Bazett	9.4 (2.5-16.5)	9.6 (3.7-16.2)	1.0
P_R_ proportion (%)			
4 ms, Wodey	67.2	68.7	1.0
4 ms, Bazett	68.0	73.9	0.17
7 ms, Wodey	53.8	51.8	1.0
7 ms, Bazett	57.5	61.5	0.64

Preload responsive (P_R_), * *p* < 0.0001 comparing baseline to during preload challenge (PC)

### Unsupervised trajectory phenotypes

**Fig. 4:**
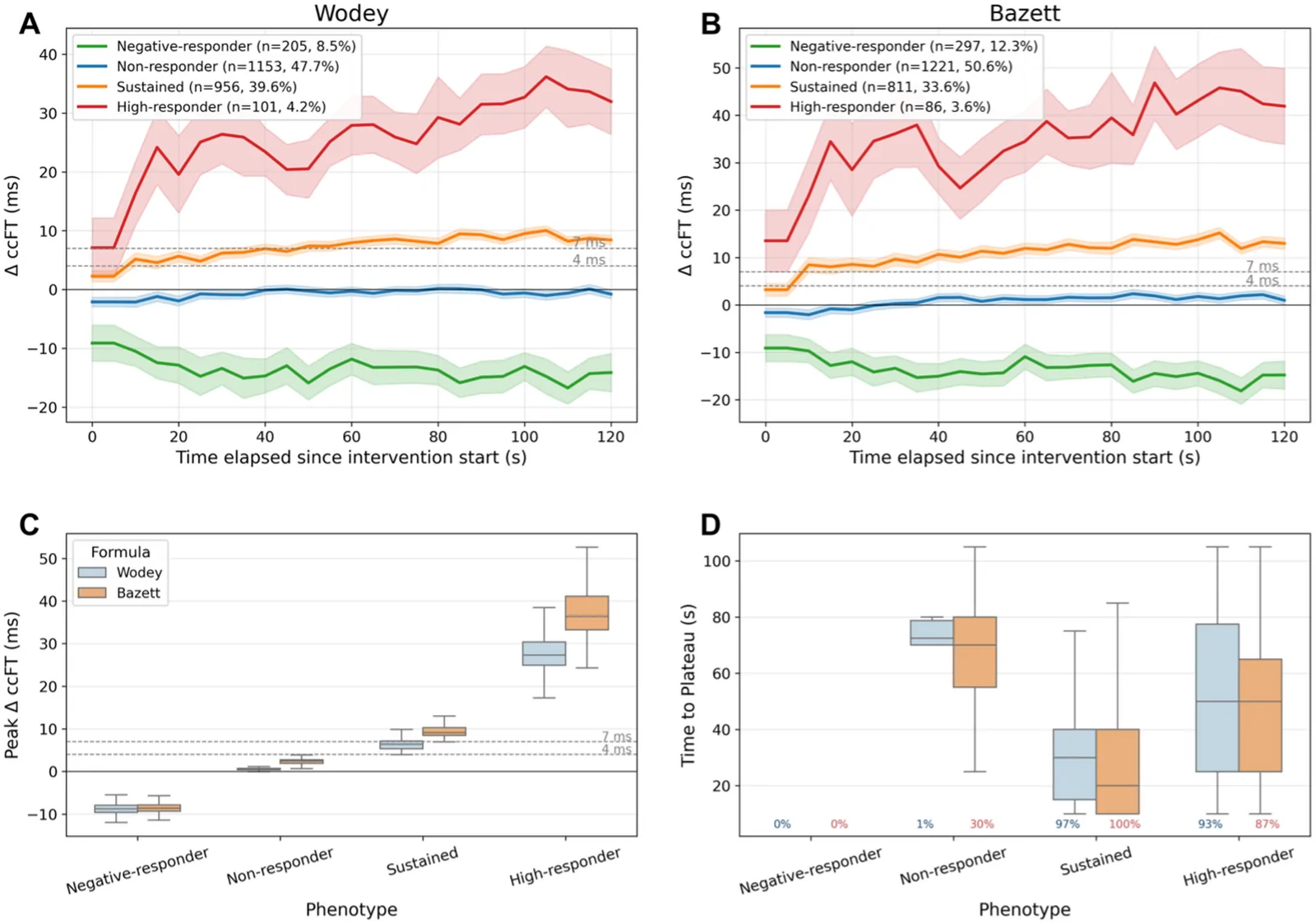
ccFT_Δ_ trajectory phenotypes identified by k-means clustering and post-hoc assessment of shape. **A** and **B** show mean trajectory for each response phenotype and 95% CI (shaded). Shown are bootstrap-derived phenotype-level peak (**C**) and time to sustained increase in ccFT_Δ_ (**D**) across both correction methods (percentages are of iterations with detectable sustained increase)

Unsupervised k-means clustering on ccFT_Δ_ trajectories agnostic to thresholding revealed four response phenotypes separately for both methods of ccFT correction in 2,415 eligible patients. The phenotypes consisted of the following: (1) Negative-responders, who exhibited a paradoxical decline into PC (Wodey 8.5%, median peak ccFT_Δ_ = − 8.8 ms; Bazett 12.3%, peak ccFT_Δ_ = − 8.6 ms) (2) Non-responders, who showed a slow rise in ccFT that predominantly did not reach a sustained increase (Wodey 47.7% of the cohort, peak ccFT_Δ_ = 0.53 ms, median TTP = 72.5; Bazett 50.6%, peak ccFT_Δ_ = 2.5 ms, TTP = 70.0 s) (3) Sustained responders, with a steady rise in ccFT (Wodey 39.6%, peak ccFT_Δ_ = 6.4 ms, TTP = 30.0 s; Bazett 33.6%, peak ccFT_Δ_ = 9.1 ms, TTP = 20.0 s) (4) and a small but pronounced High-responder phenotype (Wodey 4.2%, peak ccFT_Δ_ = 27.3 ms, TTP = 50.0 s; Bazett 3.6%, peak ccFT_Δ_ = 36.3 ms, TTP = 50.0 s) (Fig. 4).

## Discussion

To our knowledge, this is the largest dataset of ED patients with suspected sepsis receiving continuous assessment of Doppler systolic time during a preload challenge. This analysis includes 4,367 patients encompassing nearly 1 million carotid Doppler cardiac cycles. The use of an innovative wireless, wearable critical care Doppler ultrasound system made this possible, enabling us to characterize the median ccFT_Δ_, time to first sustained peak ccFT_Δ_, preload response trajectories, and the interaction between absolute, baseline ccFT and P_R_. We considered this analysis using two of the most common clinical equations for correcting the duration of systole for cycle length: Wodey and Bazett. Our observations are clinically relevant because we describe the fraction of P_R_ versus P_UR_ in ED patients with suspected sepsis based upon ccFT_Δ_, gauge how long a PC should be performed, and describe the behaviour and evolution of preload response in the early phases of resuscitation.

The median ccFT_Δ_ for all 4,128 septic ED patients was 7.29 and 9.64 ms by the equations of Wodey and Bazett, respectively. These values are clinically important when we consider what is the best threshold to identify P_R_ early in sepsis care. Barjaktarevic and colleagues evaluated a number of ccFT change thresholds to best detect a 10% change in SV using the equation of Wodey [3]. Notably, a 7 ms ccFT_Δ_ had excellent specificity (i.e., 96%), with adequate sensitivity (i.e., 69%), while a 4 ms ccFT_Δ_ had very good sensitivity and specificity (i.e., 82%, 83%, respectively). Clinically this means that early in ED care, adopting a 7 ms ccFT_Δ_ risks missing some truly responsive patients given the higher false negative rate, while a 4 ms ccFT_Δ_ minimizes false negatives at a slightly higher false positive rate. Because of this, we have previously suggested that a 4 ms ccFT_Δ_ is a clinically appropriate threshold early in care [9], so long as it is safe to administer IV fluid based upon a fluid tolerance assessment [14, 15]. Of additional interest, we found that when 20 or more consecutive cardiac cycles are averaged at baseline and during preload augmentation with trans-esophageal echocardiography as the reference standard [4], a 4 ms ccFT_Δ_ was optimal, with a sensitivity and specificity very close to that reported by Barjaktarevic et al. [3]. Similarly, using Bazett’s equation and invasive arterial line pulse contour analysis as a reference standard, Yang and colleagues recently found that 7 ms ccFT_Δ_ was the optimal threshold to detect a 10% SV augmentation. In our dataset of ED patients with suspected sepsis, a 4 versus 7 ms ccFT_Δ_ would identify 69% and 52% of patients as P_R_ by Wodey and 74% and 61% as P_R_ by Bazett. Early in septic shock, following initial fluid loading, at least two-thirds of patients are expected to be P_R_ [24–26]. Given that our patients are largely assessed whilst fluid naïve and that we generally only assess P_R_ if a patient is fluid tolerant, the 4 ms ccFT_Δ_ by Wodey’s equation is more in-line with clinical expectations.

Whether by Wodey or Bazett equation or based upon 4 or 7 ms ccFT_Δ_, the time to a sustained peak effect for P_R_ patients was at around 27.5 s into PC, although estimates were later and less precise in irregular patients. This finding has practical relevance for clinicians using Doppler ultrasound to predict P_R_ as it identifies the optimal physiological response time during preload augmentation in most patients. Notably, this temporal window is consistent with previous data where P_R_ was detected during passive leg raising and descending aortic blood flow measured by esophageal Doppler was the reference standard [27]. This concordance strengthens the plausibility that ccFT_Δ_ reflects change in left ventricular output during preload augmentation [3, 4, 28]. Furthermore, given that P_UR_ patients and the non-responder phenotypes did not see a sustained rise in ccFT within the first 60 s, the PC may safely be truncated earlier. However, for irregular rhythm patients, despite similar ccFT_Δ_ proportions, confidence intervals are much larger, implying longer PC observation periods may be required in this group. Collectively, this may streamline clinical workflow and improve dynamic assessment efficiency in busy environments like the ED.

We found a significant yet very weak relationship between baseline, absolute ccFT and P_R_ based on both thresholds and equations. In other words, a static, baseline ccFT alone is insufficient to identify ccFT_Δ_ with clinically actionable accuracy. This finding is counter to other literature, primarily in the elective, anesthesia space, where low absolute ccFT predicts P_R_ [29–33]. We suspect that in septic patients there is a higher percentage of patients with a depressed Starling curve because of acute systolic and/or diastolic dysfunction [34, 35]. Such patients can have low absolute ccFT and/or low absolute SV [36] but remain P_UR_ as previously described [11, 12, 37]. These findings emphasize the importance of performing a dynamic maneuver to elaborate the slope of the Starling curve, prior to administering preload. In other words, measuring ccFT at baseline and then again during preload modification to calculate the *change* in ccFT is critical to infer P_R_, as reported by others [3, 5, 38–41].We believe this to be especially important in acutely septic patients because in this context, targeted volume management by dynamic assessments significantly reduced mortality in a recent systematic review and meta-analysis of randomized studies [6].

Finally, in an exploratory, hypothesis-generating, sub-analysis using an unsupervised and a threshold agnostic- approach, we describe preload response that may be hidden through a binary classification snapshot based on ccFT_Δ_ thresholds. Both Wodey and Bazett equations independently recovered the same four phenotypes, which likely indicates underlying physiology rather than artifacts of heart rate correction. Indeed, for both equations, the recovered trajectories clustered away from the 4 and 7 ms thresholds and reveal a similar TTP around 20–30 s into PC for the responder phenotypes. Notably the negative-responder phenotype, defined by a paradoxical decline in beat-level ccFT_Δ_ during PC, encompassed a clinically relevant pattern consistent with a fluid-intolerant plateau as previously described [11]. This approach provided a data-driven characterization of early ccFT_Δ_ response patterns in responsive patients; whether these phenotypes are associated with patient-centered outcomes warrants further study. We think that these findings are notable given that the time course and evolution of P_R_ remain under studied [42].

Our study has several limitations of generalizability. First, this is a single center evaluation which introduces selection bias. The patients who screen for sepsis in our hospital may be different in some way (e.g., cardiovascular comorbidities, socioeconomic status, etc.) such that these findings may not generalize. Because this dataset is not linked to demographic and clinical characteristics (e.g., age, illness severity, SOFA score, mortality, etc.), it is difficult to evaluate how well this cohort represents early sepsis resuscitation and the clinical applicability of these findings. However, some of our findings imply that this population does represent a cohort of early severe sepsis and septic shock. For instance, we observed known and reported proportions of P_R_ versus P_UR_ patients [21], time to peak preload effect [27], and rates of arrhythmia/irregular rhythm [43] in these patients. Second, part of our sepsis resuscitation protocol involves nursing-led point of care ultrasound for fluid intolerance [14, 18, 19]. If it appears unsafe to administer IV fluid (i.e., the patient is fluid intolerant) by either physical examination or radiographic or ultrasonographic findings, preload responsiveness testing is often not done because an alternative strategy to augment perfusion is chosen (e.g., vasoactives). Accordingly, this analysis concentrates patients who appear euvolemic or hypovolemic in the ED and may not represent the entire spectrum of early septic pathophysiology. Third, patients with significant neck anatomical abnormalities or those who are delirious and cannot cooperate with a fluid responsiveness assessment are also excluded from carotid Doppler assessments. Fourth, patients received preload augmentation at the discretion of the treating clinician, which at our institution is Trendelenburg maneuver or a rapid fluid challenge, as described above. These are physiologically distinct maneuvers and analyzing them together limits the interpretation of time to peak estimates and trajectories. Fifth, although exploratory, the trajectory clustering is limited by edge-filling for sessions with incomplete coverage of the observation window, potentially introducing artificial flatness in late-window cluster means. Furthermore, this trajectory analysis is inherently descriptive rather than an independent validation of physiological responsiveness because no reference standard for SV_Δ_ was employed. Finally, inclusion in our sepsis protocol begins with a screening alert based upon systemic inflammatory response syndrome (SIRS) and organ dysfunction. We do not screen patients based upon Sepsis-3 criteria because, in the United States, the Centers for Medicare and Medicaid Services grounds its extraction criteria for bundle completion based upon the older sepsis definition. Screening for sepsis based upon SIRS has diminished specificity (i.e., increased false positive rates) in the ED with the goal of keeping sensitivity high (i.e., mitigating false negatives) because sepsis is a life-threatening organ dysfunction [14]. Thus, a clinically significant fraction of the patients in this analysis likely do not go on to have sepsis based upon either the older or newer Sepsis-3 definitions; because this data is de-identified, we cannot specifically comment on this proportion which is a limitation. Despite these limitations, our investigation has many strengths. To our knowledge this is the largest reported dataset of ED patients with suspected sepsis receiving P_R_ assessments by Doppler ultrasound of any kind; this analysis clarifies ccFT_Δ_ thresholds and ccFT evolution over the course of a PC. These data are important for determining length of PC which streamlines care but also forms hypotheses for forthcoming analyses linking this Doppler data to patient outcome. For instance, does the pattern of ccFT_Δ_ during a PC in the ED associate with mortality or another life-threatening organ dysfunction?

In conclusion, this study comprises nearly 1 million carotid Doppler cardiac cycles obtained from over 4,100 ED patients with suspected sepsis. In these patients, 52% and 69% are judged to be P_R_ on their first assessment based upon 7 ms and 4 ms ccFT_Δ_ thresholds, respectively. The time-to-peak ccFT_Δ_ is in the first 30 s of intervention for most responsive patients in the overall cohort, consistent with passive leg raise data and esophageal Doppler data. Four distinct and heretofore undescribed ccFT_Δ_ trajectories during a PC are reported and may represent distinct preload response patterns during early suspected sepsis resuscitation.

## Supplementary Information

Supplementary Material 1.

## Data Availability

Upon reasonable request

## References

[1] Kenny J-ES (2025) A framework for flow time measured by Doppler ultrasound. Ultrasound J 17(1):10 10.1186/s13089-025-00414-8 PMC11751365 39836314

[2] van Houte J, Eerdekens R, Manning F, Te Pas M, Houterman S, Wijnbergen I, Montenij L, Tonino P, Bouwman A (2024) Is the Corrected Carotid Flow Time a Clinically Acceptable Surrogate Hemodynamic Parameter for the Left Ventricular Ejection Time? Ultrasound Med Biol 50(4):528–535 10.1016/j.ultrasmedbio.2023.12.013 38242742

[3] Barjaktarevic I, Toppen WE, Hu S, Montoya EA, Ong S, Buhr R, David IJ, Wang T, Rezayat T, Chang SY (2018) Ultrasound Assessment of the Change in Carotid Corrected Flow Time in Fluid Responsiveness in Undifferentiated Shock. Crit Care Med 11:1040–1046 10.1097/CCM.0000000000003356 PMC6774608 30134304

[4] Kenny JS, Clarke G, Atwi S, Kerrebijn I, Savery T, Knott M, Munding CE, Elfarnawany M, Eibl AE, Eibl JK et al (2025) Carotid Artery Corrected Flow Time Measured by Wearable Doppler Ultrasound Detects Stroke Volume Change Measured by Trans-Esophageal Echocardiography Following Coronary Artery Bypass Grafting. CHEST Crit Care 3:100138

[5] Yang Y, Li M, Yang C, Shi Z, Wu H, Chen G, Chen L (2025) Comparison of the change in carotid corrected flow time and stroke volume variation for assessing volume responsiveness in general anesthesia patients: a prospective, observational study. J Clin Monit Comput 40:429–438 10.1007/s10877-025-01375-y PMC13053328 41160382

[6] Wang J, Blake LM, Orozco N, Fiorini K, McChesney C, Slessarev M, Prager R, Leligdowicz A, Sharif S, Lewis K et al (2025) Dynamic Measures of Fluid Responsiveness to Guide Resuscitation in Patients With Sepsis and Septic Shock: A Systematic Review and Meta-Analysis. Crit Care Explorations 7(9):e1303 10.1097/CCE.0000000000001303 PMC12440473 40953281

[7] Messina A, Grieco DL, Alicino V, Matronola GM, Brunati A, Antonelli M, Chew MS, Cecconi M (2025) Assessing fluid responsiveness by using functional hemodynamic tests in critically ill patients: a narrative review and a profile-based clinical guide. J Clin Monit Comput 39(3):481–493 10.1007/s10877-024-01255-x 39831948

[8] Kenny J-É, Munding CE, Eibl JK, Eibl AM, Long BF, Boyes A, Yin J, Verrecchia P, Parrotta M, Gatzke R (2021) A novel, hands-free ultrasound patch for continuous monitoring of quantitative Doppler in the carotid artery. Sci Rep 11(1):1–11 10.1038/s41598-021-87116-y PMC8032670 33833288

[9] Kenny JS, Gibbs SO, Johnston D, Yang Z, Hofer LM, Elfarnawany M, Eibl JK, Johnson A, Buecker AJ, Lau VC et al (2023) The time cost of physiologically ineffective intravenous fluids in the emergency department: an observational pilot study employing wearable Doppler ultrasound. J Intensive Care 11(1):7 10.1186/s40560-023-00655-6 PMC9933329 36793079

[10] Kenny J-É, Barjaktarevic I, Mackenzie DC, Rola P, Haycock K, Eibl AM, Eibl JK (2021) Inferring the Frank–Starling Curve From Simultaneous Venous and Arterial Doppler: Measurements From a Wireless, Wearable Ultrasound Patch. Front Med Technol 3:1–7 10.3389/fmedt.2021.676995 PMC8757733 35047930

[11] Kenny JS, Gibbs SO, Eibl JK, Eibl AM, Yang Z, Johnston D, Munding CE, Elfarnawany M, Lau VC, Kemp BO et al (2023) Simultaneous venous-arterial Doppler during preload augmentation: illustrating the Doppler Starling curve. Ultrasound J 15(1):32 10.1186/s13089-023-00330-9 PMC10382420 37505318

[12] Kenny JS, Prager R, Rola P, Haycock K, Gibbs SO, Johnston DH, Horner C, Eibl JK, Lau VC, Kemp BO (2024) Simultaneous venous-arterial Doppler ultrasound during early fluid resuscitation to characterize a novel Doppler starling curve: a prospective observational pilot study. J Intensive Care Med 39(7):628–635 10.1177/08850666231224396 PMC11188059 38190576

[13] Kenny JS, Rola P (2024) Optimising flow without congestion using the venous-arterial Doppler enhanced resuscitation framework. Australas J Ultrasound Med 27(3):193–196 10.1002/ajum.12388 PMC11423439 39328256

[14] Nunnally J, Ko SM, Ugale K, Lowe T, Bond J, Kenny J-ES, Fargo RA, Haycock K (2024) A nursing-led sepsis response team guiding resuscitation with point-of-care ultrasound: A review and model for improving bundle compliance while individualizing sepsis care. SAGE Open Med 12:20503121241290378 10.1177/20503121241290378 PMC11549707 39526100

[15] Kaufman CS, Wu D, Eibl JK, Kenny J-ES (2026) Ultrasonographic Measures of Safe and Fluid Effective Resuscitation (SaFER): Incorporating the VExUS and VTI into Protocolized Care. SN Compr Clin Med 8(1):73

[16] Selden N, Skaggs H, Lowe T, Haycock K, Dinh V (2017) 338 Assessing the Utility of Nursing-Performed Point-of-Care Ultrasound as a Guide to Fluid Resuscitation of Septic Patients in the Emergency Department. Ann Emerg Med 70(4):S134

[17] Kalam S, Selden N, Haycock K, Lowe T, Skaggs H, Dinh VA (2023) Evaluating the Effect of Nursing-Performed Point-of-Care Ultrasound on Septic Emergency Department Patients. Cureus 15(6):e40519 10.7759/cureus.40519 PMC10350309 37461778

[18] Kenny J-ES (2022) Assessing Fluid Intolerance with Doppler Ultrasonography: A Physiological Framework. Med Sci 10(1):12 10.3390/medsci10010012 PMC8883898 35225945

[19] Kattan E, Castro R, Miralles-Aguiar F, Hernández G, Rola P (2022) The emerging concept of fluid tolerance: A position paper. J Crit Care 71:154070 10.1016/j.jcrc.2022.154070 35660844

[20] Kenny J-ES, CORRECTING THE CAROTID FLOW TIME WITH THE FORMULA OF BAZETT (2025) MIND THE UNITS. Shock 63(1):176–177 10.1097/SHK.0000000000002487 39297654

[21] Messina A, Dell’Anna A, Baggiani M, Torrini F, Maresca GM, Bennett V, Saderi L, Sotgiu G, Antonelli M, Cecconi M (2019) Functional hemodynamic tests: a systematic review and a metanalysis on the reliability of the end-expiratory occlusion test and of the mini-fluid challenge in predicting fluid responsiveness. Crit Care 23(1):264 10.1186/s13054-019-2545-z PMC6664788 31358025

[22] Barthélémy R, Kindermans M, Delval P, Collet M, Gaugain S, Cecconi M, Mebazaa A, Chousterman BG (2021) Accuracy of cumulative volumes of fluid challenge to assess fluid responsiveness in critically ill patients with acute circulatory failure: a pharmacodynamic approach. Br J Anaesth 128(3):236–243 10.1016/j.bja.2021.10.049 34895718

[23] Minini A, Abraham P, Malbrain M (2020) Predicting fluid responsiveness with the passive leg raising test: don’t be fooled by intra-abdominal hypertension! Ann Transl Med 8(12):799 10.21037/atm.2019.12.14 PMC7333095 32647724

[24] Hernández G, Ospina-Tascón GA, Damiani LP, Estenssoro E, Dubin A, Hurtado J, Friedman G, Castro R, Alegría L, Teboul J-L (2019) Effect of a resuscitation strategy targeting peripheral perfusion status vs serum lactate levels on 28-day mortality among patients with septic shock: the ANDROMEDA-SHOCK randomized clinical trial. JAMA 321(7):654–664 10.1001/jama.2019.0071 PMC6439620 30772908

[25] Kattan E, Ospina-Tascón GA, Teboul J-L, Castro R, Cecconi M, Ferri G, Bakker J, Hernández G, Investigators A-S (2020) Systematic assessment of fluid responsiveness during early septic shock resuscitation: secondary analysis of the ANDROMEDA-SHOCK trial. Crit Care 24(1):23 10.1186/s13054-020-2732-y PMC6979284 31973735

[26] The ANDROMEDA-SHOCK-2 Investigators for the ANDROMEDA Research Network SSoA, Reanimation P, Therapy, Network LAIC (2025) Personalized Hemodynamic Resuscitation Targeting Capillary Refill Time in Early Septic Shock: The ANDROMEDA-SHOCK-2 Randomized Clinical Trial. JAMA 334(22):1988–1999 10.1001/jama.2025.20402 PMC12573117 41159835

[27] Monnet X, Rienzo M, Osman D, Anguel N, Richard C, Pinsky MR, Teboul J-L (2006) Passive leg raising predicts fluid responsiveness in the critically ill. Crit Care Med 34(5):1402–1407 10.1097/01.CCM.0000215453.11735.06 16540963

[28] Kerrebijn I, Atwi S, Horner C, Elfarnawany M, Eibl AM, Eibl JK, Taylor JL, Kim CH, Johnson BD, Kenny JS (2023) Correlation between changing carotid artery corrected flow time and ascending aortic Doppler flow velocity. Br J Anaesth 131(6):e192–e195 10.1016/j.bja.2023.09.016 37863770

[29] Singla D, Gupta B, Varshney P, Mangla M, Walikar BN, Jamir T (2023) Role of carotid corrected flow time and peak velocity variation in predicting fluid responsiveness: a systematic review and meta-analysis. Korean J Anesthesiol 76(3):183–193 10.4097/kja.22385 PMC10244609 36377330

[30] Kim D-H, Shin S, Kim N, Choi T, Choi S, Choi Y (2018) Carotid ultrasound measurements for assessing fluid responsiveness in spontaneously breathing patients: corrected flow time and respirophasic variation in blood flow peak velocity. Br J Anaesth 121(3):541–549 10.1016/j.bja.2017.12.047 30115251

[31] Jung S, Kim J, Na S, Nam WS, Kim D-H (2021) Ability of Carotid Corrected Flow Time to Predict Fluid Responsiveness in Patients Mechanically Ventilated Using Low Tidal Volume after Surgery. J Clin Med 10(12):2676 10.3390/jcm10122676 PMC8234831 34204523

[32] Lipszyc AC, Walker SCD, Beech AP, Wilding H, Akhlaghi H (2024) Predicting Fluid Responsiveness Using Carotid Ultrasound in Mechanically Ventilated Patients: A Systematic Review and Meta-Analysis of Diagnostic Test Accuracy Studies. Anesth Analg 138(6):1174–1186 10.1213/ANE.0000000000006820 38289868

[33] Wang H, Chen W, Cheng H, Liu C, Yao W, Ding F, Wang Y, Chen Y (2022) Value Of Corrected Flow Time In Common Carotid Artery In Predicting Volume Responsiveness Under Mechanical Ventilation. Shock 58(1):28–33 10.1097/SHK.0000000000001959 PMC9415202 35904148

[34] Beesley SJ, Weber G, Sarge T, Nikravan S, Grissom CK, Lanspa MJ, Shahul S, Brown SM (2018) Septic Cardiomyopathy. Crit Care Med 46(4):625–634 10.1097/CCM.0000000000002851 29227368

[35] Messina A, Vieillard–Baron A (2025) How we could use critical care echocardiography in the assessment of and management of cardiovascular phenotypes in septic shock: the good, the bad, and the ugly profiles. Intensive Care Med 51(2):397–400 10.1007/s00134-025-07782-8 39833496

[36] Cheong I, Merlo PM, Tamagnone FM (2025) Correlation between corrected carotid flow time and left ventricular outflow tract velocity-time integral using a novel technique. J Clin Ultrasound 53(1):97–102 10.1002/jcu.23838 39291774

[37] Kenny J-ES (2024) Passive leg raising uncovers venous congestion: dynamic fluid intolerance and the Doppler Starling curve. Crit Care 28(1):388 10.1186/s13054-024-05171-z PMC11590204 39587637

[38] Kimura A, Suehiro K, Juri T, Tanaka K, Mori T (2021) Changes in corrected carotid flow time induced by recruitment maneuver predict fluid responsiveness in patients undergoing general anesthesia. J Clin Monit Comput 36(4):1069–1077 10.1007/s10877-021-00736-7 34191254

[39] Jelic T, Chenkin J (2023) Carotid Flow Time Compared with Invasive Monitoring as a Predictor of Volume Responsiveness in ICU patients. Pocus j 8(2):212–216 10.24908/pocus.v8i2.16545 PMC10723903 38106371

[40] Jalil B, Thompson P, Cavallazzi R, Marik P, Mann J, El Kersh K, Guardiola J, Saad M (2017) Comparing Changes in Carotid Flow Time and Stroke Volume Induced by Passive Leg Raising. Am J Med Sci 355(2):168–173 10.1016/j.amjms.2017.09.006 29406045

[41] Suehiro K (2026) Carotid flow time: a new surrogate for fluid responsiveness? J Anesth (online ahead of print) 10.1007/s00540-026-03713-z 41832281

[42] Castro R, Kattan E, Melo P, Amthauer M, Hernandez G (2026) The time course of fluid responsiveness. Ann Intensiv Care 16:100036 10.1016/j.aicoj.2026.100036 PMC13045553 41938903

[43] Walkey AJ, Wiener RS, Ghobrial JM, Curtis LH, Benjamin EJ (2011) Incident Stroke and Mortality Associated With New-Onset Atrial Fibrillation in Patients Hospitalized With Severe Sepsis. JAMA 306(20):2248–2254 10.1001/jama.2011.1615 PMC3408087 22081378

