# Supplementary material for: The effect of preload on Doppler systolic time in a large emergency department cohort with suspected sepsis
Source: Intensive Care Med Exp. 2026 Oct 12;14:135. doi: 10.1186/s40635-026-00987-1 (PMC13658765; doi:10.1186/s40635-026-00987-1)
Supplement: Supplementary file 1 [file 40635_2026_987_MOESM1_ESM.docx]

**Supplementary Information**

**Supplementary Figure 1: Distribution of within patient ccFT_Δ_ using Wodey and Bazett equations.** Both methods showed positive, above 7ms median change. Both distributions were positively skewed and heavy-tailed, indicating between-patient variability. Dotted lines indicate the mean, median, and 4 ms and 7 ms response thresholds.

**B**

**A**

**Supplementary Figure 2. Patient-level variability in ccFTΔ trajectories during preload challenge.** Temporal trajectories of ccFTΔ during preload responseive (PR; circles) and preload-unresponsive (PUR; squares) patients based on ≥4 ms (A) and ≥7 ms (B) response thresholds for the Wodey (blue) and Bazett (red) correction methods. Cardiac-cycle ccFTΔ values were averaged across 5-second time bins within each patient. Lines represent the median of these patient-level values and shaded regions represent the interquartile range (25th–75th percentile) across patients. Vertical dotted lines indicate the median time-to-peak (TTP) ccFTΔ in responsive patients, with vertical shading representing the bootstrap-derived 95% confidence interval for TTP.
